# “I am Young, Why Should I Vaccinate?” How empathetic and aggressive communication on social media impact young adults’ attitudes toward COVID-19 vaccination

**DOI:** 10.3389/fpubh.2023.1190847

**Published:** 2023-10-06

**Authors:** Jaroslava Kaňková, Melanie Saumer, Ariadne Neureiter, Sofia Darovskikh, Elena Shargina, Jörg Matthes

**Affiliations:** Advertising and Media Psychology Research Group, Department of Communication, University of Vienna, Vienna, Austria

**Keywords:** COVID-19, vaccination support, health communication, expectancy violation, psychological distance, credibility, communication style

## Abstract

**Introduction:**

To combat the current COVID-19 pandemic, high vaccination rates are of crucial value. However, young people in particular tend to be hesitant toward vaccination. On social media, young adults are often called to vaccinate in an aggressive tone, arguing that there is no choice than to vaccinate and that all else is wrong.

**Methods:**

In an experimental study (*N* = 410), we investigated the effects of (a) empathetic vs. aggressive communication styles in social media postings and (b) the origin of the communicator on young adults’ supportive attitudes toward COVID-19 vaccinations. We treated the gender of the communicator as a moderator, and expectancy violation, psychological distance as well as the perceived credibility of the communicator as mediators.

**Results:**

Findings showed that an aggressive communication style generally had a negative impact on young adults’ COVID-19 vaccination attitudes, fully mediated by expectancy violation and perceived credibility of the communicator. Gender and the origin of the communicator did not moderate this mediation processes.

**Discussion:**

Further implications for online health communication strategies are discussed.

## 1. Introduction

Young adults in particular often hesitate when it comes to the COVID-19 vaccination (1). Although it seems necessary to motivate young adults to get vaccinated against COVID-19 to combat the pandemic, it is neither easy to reach them in order to draw their attention to vaccinations, nor to encourage them to get vaccinated [e.g., (2, 3)]. Since social media is an important source for young adults to retrieve information about the COVID-19 pandemic [e.g., (3)], it could also influence young adults’ health-related attitudes and behavior. Indeed, prior research has shown that communicators on social media such as health experts can motivate young adults to either get vaccinated (4–7) or to refrain from vaccination—possibly resulting in so-called vaccination hesitancy [e.g., (8)]. Whether one or the other occurs might depend on how health experts communicate about COVID-19 vaccinations on social media [e.g., (9)].

This paper focuses on two communication styles commonly used on social media concerning COVID-19: Aggressive and empathic messages. The former is characterized by uncivil language, attacks, or general unfriendliness toward opposition [e.g., (10)] and has recently become prevalent in vaccination debates on social media. The United Nations even refer to a wave of aggressive communication in form of hate speech—offline and online—due to COVID-19 [e.g., (11)], possibly leading to less persuasion effects than empathetic communication [e.g., (12)] characterized by verbal affirmation, experience sharing, and emotional reactivity (13). However, previous studies have demonstrated that aggressive communication style can also generate positive outcomes under certain circumstances (14, 15).

Based on earlier research focusing on general vaccine hesitancy, it can be assumed that besides the communication style, other characteristics of the health expert might influence individuals’ support for the COVID-19 vaccinations [e.g., (16)]. For instance, when individuals’ expectancies regarding communication style and gender-based assumptions about the health experts get violated, the credibility of health experts could suffer, possibly resulting in less support for the COVID-19 vaccinations [e.g., (16–19)]. Additionally,—independently of the health experts’ communication style on social media—international origin of the health experts could heighten individuals’ perceptions of psychological distance toward them. This could make the health experts less credible and thus, less supportive attitudes toward COVID-19 vaccinations could result [e.g., (20, 21)].

However, although knowledge about appropriate communication styles of health experts on social media regarding COVID-19 vaccines is increasingly important to combat COVID-19 (22), until yet to the best of the authors’ knowledge, no study has focused on the impact of health experts’ communication style on social media on young adults’ COVID-19 vaccination attitudes. Additionally, neither the health experts’ gender, nor their origin has been taken into account when looking at the effects of their online messages on young adults’ supportive COVID-19 vaccination attitudes. Therefore, the aim of the proposed study is to investigate how the communication style of the health expert, the health experts’ gender as well as their origin is contributing to young adults’ supportive attitudes toward COVID-19 vaccinations [e.g., (14, 20)].

## 2. Health experts’ communication style on social media

In the case of COVID-19, health experts increasingly use social media to inform individuals’ about COVID-19 [e.g., (3)]. While some of them tend to communicate rather aggressively, others might use more empathetic ways to educate about COVID-19 [e.g., (11, 14)].

An *aggressive communication style* is characterized by hostility, offensive language, attacks, or unfriendly tonalities [e.g., (10)]. During the COVID-19 pandemic, scientific communication became more aggressive [e.g., (14)]. A number of health experts and scientists became frustrated with people who discredited science and refused to follow scientific recommendations. This has led to the use of aggressive language by the scientific community (14). Research has demonstrated that aggressive communication can lead to increased polarization, decreased persuasion, and reinforcement of previously held attitudes [e.g., (12)]. Moreover, aggressive communication can negatively affect trust in communicators, their likeability, and the perceived quality of the message [e.g., (15, 23)]. Within the specific context of scientific debate, an aggressive communication tone was perceived as less trustworthy, less credible and individuals felt like they learned less from the debate (24).

In contrast, an *empathetic communication style* is characterized by verbal affirmation, experience sharing, and emotional reactivity (13). Regarding COVID-19 online communication, health experts and scientists are increasingly advised to use empathetic communication styles to signal users that their concerns are understood and that they can trust their messages (25). Incorporating empathy into online communication has yielded positive results when it comes to achieving the goals that the communication was aimed at [e.g., (26)]. Further, research has shown that empathetic communication in the context of COVID-19 leads to more positive attitudes toward COVID-19 vaccination by young adults through not only feeling that their vaccination concerns are understood, but also through heightened trust in the communicator [e.g., (27)].

### 2.1. Health experts’ gender and expectancy violation

Gender (and related stereotypical beliefs) is one of the factors on which people base their expectations of how others are going to communicate and behave [e.g., (18)]. Prior research has shown that men tend to be more verbally aggressive than women. Further, men tend to argue more and avoid arguments less [e.g., (28)]. In contrast, previous research has demonstrated that women tend to be more empathetic and more capable of expressing empathy than men [e.g., (19)]. Additionally, women are also stereotypically portrayed as more nurturing and empathetic, whereas men are displayed as less emotional (29).

The Expectancy Violation Theory (EVT) (30) suggests that once expectations are violated in communication, cognitive arousal is initiated, prompting attitude and behavior changes. Based on this, it can thus be expected that violations of young adults’ expectations caused by the communication style of the health experts online (either empathetic or aggressive) will be moderated by gender. This argument is in line with the work of Burgoon and Miller (31), who suggest that aggressive communication is expected and tends to be more effective when used by male speakers. However, when females employ an aggressive communication style, it violates recipients’ expectations, thereby undermining the effectiveness of their persuasion efforts. Similarly, in the context of health communication, female physicians are expected to communicate in a non-aggressive manner, whereas this expectation does not apply to male physicians (32, 33). In line with this, both male and female communicators have the potential to contradict audience expectations either by being aggressive (in the case of females) or by not being aggressive (in the case of males) (18). Drawing on the EVT (30), we assume that expectancies aligned with gender stereotypes will be violated if a female health expert communicates aggressively about COVID-19 vaccinations, as clearly demonstrated by the aforementioned previous research. Further, following the same thought, a male health expert communicating in an empathetic way about COVID-19 vaccination concerns, will lead to increase in expectancy violation. Furthermore, earlier research has also emphasized that message recipients’ own gender does not impact the gender-based expectations about the source [e.g., (18, 32)]. We thus hypothesize the following:

*H1*: An aggressive message by a female will lead to higher expectancy violation compared to the same message by a male.

*H2*: An empathetic message by a male will lead to higher expectancy violation compared to the same message by a female.

### 2.2. Expectancy violation and perceived source credibility

One core assumption of persuasion research is that the characteristics of the message source have a significant impact on the persuasion outcomes [e.g., (34)]. Hovland et al. (35) introduced the term *source credibility* which encompasses both trustworthiness and expertise of the source. Several studies have demonstrated that highly credible sources lead to more positive persuasion outcomes than less credible sources [e.g., (34, 36)] indicating that communicators with high levels of credibility are superior in achieving persuasion effects compared to communicators with low credibility levels [e.g., (16)].

However, recipients’ expectancy violation might influence the level of credibility of a communicator [e.g., (30)]. For instance, Bullock and Hubner (17) suggested that the use of informal language by politicians on social media violates the expectations of the audience—since such authorities are expected to appear serious and competent—, and leads to a decrease in their credibility. This also applies to health professionals [e.g., (37)]. In the context of COVID-19 health experts, this would mean that when communicators violate young adults’ expectancies, for instance by using an unexpected communication style, their credibility might suffer as a consequence. Thus, we derive the following hypothesis:

*H3*: Expectancy violation is negatively related to perceived credibility of the communicator.

## 3. Health experts’ origin and psychological distance

In the context of COVID-19 online communication, both, local and international health experts speak up on a regular basis using social media platforms to inform individuals about health measures to combat the pandemic [e.g., (38)]. In light of scientific internationality—that a pandemic brings—the concept of psychological distance becomes important. Based on the Theory of Psychological Distance [e.g., (20)], psychological distance refers to “objects (…) that are not present in the direct experience of reality” (20, p. 353). Thus, international health experts might be perceived by recipients’ as spatially further away, less similar to themselves [e.g., (39)], and thus, more psychological distant than local health experts. Although, psychological distance has been researched within the context of perceptions of virus origins [e.g., (40)], potential effects of individuals’ perceptions of psychological distance toward online communicators, such as health experts on social media, are not studied in the pandemic context until now. Thus, based on the Theory of Psychological Distance [e.g., (20)], we assume that higher spatial distance—in terms of internationality—creates a higher psychological distance between the communicator and the audience, independent of which communication style is used. We hence hypothesize as follows:

*H4*: An (a) aggressive and (b) empathetic message by an international communicator will lead to higher psychological distance compared to the same message by a local one.

### 3.1. Psychological distance and perceived source credibility

Simons et al. (21) suggested that communicators who are perceived from their audience as similar to themselves, are more likely to be respected, perceived as more attractive, more trusted, and have a better ability to change the audiences’ attitudes in comparison to communicators who are perceived from their audience as dissimilar to themselves. Further, perceptions of similarity with the communicator are influenced by perceived psychological distance to the communicator (21). Since perceived similarity with a communicator is positively related to perceptions of message credibility [e.g., (41)], we argue, that message credibility might also be influenced by perceptions of psychological distance. Thus, in the context of this study, young adults’ perception of psychological distance to the health expert could dampen the credibility of the message. Along these lines, an international health expert communicating about COVID-19 on social media might be perceived by young adults as less credible than a local one. Thus, we propose the following hypothesis:

*H5*: Psychological distance is negatively related to perceived credibility of the communicator.

## 4. Perceived source credibility and support for the COVID-19 vaccination

Credibility of the communicator is considered as one of the characteristics that can significantly influence individuals’ attitudes or their behavioral intentions, such as positive vaccination attitudes and intentions to get vaccinated [e.g., (42)]. Additionally, perceiving a communicator as credible source of information leads to stronger persuasion effects [e.g., (16)]. In contrast, negative attitudes toward media messages by officials, such as health experts that try to motivate individuals to vaccinate against COVID-19, often occur due to lack of source credibility (17). In line with this, we argue that young adults’ perceived credibility of the communicating health expert might decrease widespread doubt and mistrust toward the benefits that vaccines can provide [e.g., (43, 44)] and positively influence young adults’ supportive attitudes toward COVID-19 vaccination [e.g., (45)]. We therefore derive the following hypothesis:

*H6*: Perceived credibility of the communicator is positively related to supportive attitudes toward COVID-19 vaccination.

For our overall conceptual model, please see Supplementary Figure 1.

## 5. Methods

We carried out a 2 × 2 × 2 between-subject design experiment. We manipulated the communication style (aggressive vs. empathetic) of fictitious health experts that post information about the COVID-19 vaccination on the social media platform Twitter, which has been used most consistently for dissemination of information by the medical community [e.g., (46)]. Furthermore, the communicators’ gender (female vs. male), and the origin of the communicator (local workplace affiliation in Australia vs. international workplace affiliation at the World Health Organization) was manipulated.

The data collection took place between October 6 and October 20, 2021. This study was part of a bigger project in Australia involving independent experiments with different and unrelated topics. The whole project was ethically approved by the Institutional Review Board of the University of Vienna (approval ID: 20210714_055).

### 5.1. Participants

Subjects (*N* = 410) were recruited with the help of a professional market research institute. The sample consisted of young adults aged 16–26 years (*M*_age_ = 21.06, *SD_age_* = 2.91) living in Australia. While the gender of participants was approximately equally distributed in the sample (57.8% female; 42.2% male), participants came from diverse educational backgrounds (6.3% no formal education, 12% complete lower-secondary education, 30.7% complete upper-secondary education, 12.7% complete post-secondary, non-tertiary education, 6.3% complete short cycle tertiary education, 22.9% complete bachelors or equivalent level degree, 7.1% complete masters or equivalent level degree, and 2.0% complete doctoral or equivalent level degree).

### 5.2. Stimuli

Our study employed a multi-message design, involving two distinct Twitter profiles for each of the eight experimental conditions. Each participant was exposed to a total of five tweets: three from one profile and two from the other. All tweets contained the same information about why getting the COVID-19 vaccination is important, only the wording tone was adjusted to the respective experimental condition (aggressive vs. empathetic; please see Supplementary Data). More precisely, we created the posting text based on three verbal factors that are said to enhance perceived empathy, namely *verbal affirmation, experience sharing,* and *emotional reactivity* (13). The empathetic messages contained a sympathizing way of convincing people to get the COVID-19 vaccination by addressing that having “doubts” and “distress” is totally relatable and occurred to the communicator as well. Since aggressive online messages usually include elements of hostility, offensive language, attacks or unfriendly tone [e.g., (10)], for the aggressive postings, we employed a rather aggressive tone urging people to get vaccinated, otherwise they would be “ignorant,” “irresponsible,” “selfish,” “blind” and “wrong.”

To incorporate the further manipulations of the communicators’ gender and origin, we varied the names (male vs. female), used male vs. female stock photos and implemented local vs. international workplace affiliations that were depicted in the Twitter profile header. Otherwise, the design of the different Twitter profiles was consistently created in the same way for each profile and condition (see Supplementary Data).

To demonstrate the expertise of the Twitter users giving advice specifically on COVID-19 vaccinations, they were described as either “epidemiologist” or “immunologist.” To account for recognition effects, we chose fictitious profiles with made up names and stock photos.

### 5.3. Measures

#### 5.3.1. Mediators

All items, reliability scores (Cronbach’s Alpha and its 95% confidence interval, see (47)), means, and standard deviations of the scales are depicted in Supplementary Table 1.

For *expectancy violation,* participants assessed the appropriateness of the communication style that was used in the postings by rating four items adapted from Chu et al. (14), and Yuan et al. (15) on a scale from 1—“disagree strongly” to 5—“agree strongly.”

To measure *psychological distance*, we asked respondents to rate four items adapted from McCroskey et al. (48) measuring perceived similarity with the communicator on a scale ranging from 1—“disagree strongly” to 5—“agree strongly.” Afterward, we reverse coded the items so that higher values indicated more distance.

For *perceived credibility of the communicator,* we asked participants to assess nine adjective pairs regarding their attitude toward the communicator which were presented as a five-point semantic differential in the following three subdimensions: Warmth, competence, and trustworthiness. The items were adapted from Chu et al. (14), Hendriks et al. (49), and Yuan et al. (15).

#### 5.3.2. Dependent variable

We measured *supportive attitudes toward COVID-19 vaccination* with three customized items on a scale from 1—“disagree strongly” to 5—“agree strongly,” similar to Petersen et al. (50).

#### 5.3.3. Controls

Besides participants’ age, gender and education, we controlled for *political orientation*, *vaccination status* and *prior COVID-19 infection experiences*.

### 5.4. Statistical model

For our statistical analysis, we employed path analysis by using AMOS (51). We treated communication style (dummy coded), and origin of the communicator (dummy coded) as independent variables. While we treated gender of the communicator (dummy coded) as our moderator, we used expectancy violations, psychological distance, and credibility of the communicator as our mediators. As dependent variable, we added supportive attitudes toward COVID-19 vaccination.^1^

### 5.5. Randomization and manipulation check

Participants were distributed across all eight experimental conditions, with group sizes ranging from 45 to 60 participants (see Supplementary Table 2). We found no systematic differences for our control variables age, gender, education, political orientation and prior COVID-19 infections between our conditions, except for vaccination status. Further, the manipulation was deemed as successful. For more details, please see Supplementary Tables 3, 4.

## 6. Results

For all results of our model, please see Supplementary Table 5. For an overview of the state of our hypotheses, please see Supplementary Table 6.

Regarding our H1 suggesting that an aggressive message by a female health expert will lead to higher expectancy violation of young adults compared to the same message by a male, results indicated that the hypothesis is not supported. Interestingly, our analysis showed that communication style had a large direct effect on expectancy violation, independently of the gender of the communicator (*b* = 0.56, *SE* = 0.13, *p* < 0.001). Hence, H1 is not confirmed.

Similarly, our H2 stating that an empathetic message by a male health expert will lead to higher expectancy violation compared to the same message by a female, is not supported by the results. Based on our results, we conclude that there is no evidence of gender having a moderating effect on the relationship between communication style and expectancy violation (Communication style * communicator gender: *b* = −0.03, *SE* = 0.14, *p* = 0.831).

H3 suggests that expectancy violation is negatively related to credibility of the communicator, which can be confirmed. Results indicated a negative association between perceptions of expectancy violation and perceptions of the credibility of the communicator, with the effect size being medium (*b* = −0.44, *SE* = 0.05, *p* < 0.001).

Contrasting with our H4—assuming that an (a) aggressive and (b) empathetic message by an international expert will be associated with higher psychological distance compared to a local one—results showed that there is no significant interaction effect of communication style (aggressive vs. empathic) and the origin of the expert (local vs. international) on perceived psychological distance (Communication style * nationality expert: *b* = 0.30, *SE* = 0.19, *p* = 0.105). Thus, H4 is not supported.

With regard to H5 suggesting that psychological distance is negatively related to perceived credibility of the health expert, results showed that this hypothesis can be confirmed: based on our analysis, the evidence suggest a significant negative association between psychological distance and perceived credibility of the health expert, with a medium effect size (*b* = −0.37, *SE* = 0.04, *p* < 0.001).

Finally, H6 states that perceived credibility of the health expert is positively related to supportive attitudes toward COVID-19 vaccinations. Since the results showed a positive association between perceived credibility of the health expert and increased supportive attitudes toward COVID-19 vaccinations, we found support for H6 (*b = −0.28, SE = 0.06, p* < 0.001). The effect size was medium.

## 7. Discussion

The aim of this study was to investigate potential effects of health experts’ empathetic and aggressive communication styles about COVID-19 vaccinations on social media on young adults’ supportive attitude toward COVID-19 vaccination. Motivating young adults for health-related behavior such as vaccinations via effective social media campaigns is a highly relevant matter in current health communication studies [e.g., (53, 54)]. However, so far, previous research on vaccination motivation and intentions has neglected younger generations’ response to social media vaccination appeals. To combat COVID-19 by creating herd immunity, everyone should get vaccinated independently of their age. Yet, young adults—when healthy—are statistically less at risk of experiencing severe health consequences from contracting COVID-19 (3) which is reflected in young adults’ rather low willingness to get vaccinated [e.g., (2, 55)]. Since younger generations are best reached by social media campaigns, social media could be a fruitful platform for health experts to inform and motivate young adults to combat COVID-19 by getting vaccinated [e.g., (3)]. Indeed, the emergence of COVID-19 has spurred health professionals to increasingly utilize social media platforms, particularly Twitter, to expedite the dissemination of critical information regarding a novel and highly contagious disease [e.g., (56)]. Thus, the present study contributes to knowledge about the persuasive effects of online health information and the ways health experts should communicate to young adults in order to mobilize them to combat the current COVID-19 pandemic by getting vaccinated. Also, communicator’ characteristics such as the gender or the origin of the health expert, and young adults’ perceived psychological distance toward the health expert as well as their perceived credibility are of interest when investigating persuasion effects in the online context.

Our first hypothesis postulated an increasing level of young adults’ expectancy violations when the communication style is empathetic and the gender of the communicator is male, compared to when a female is communicating in an empathetic way. This could not be supported. However, it can be inferred, based on the results of our and previous research, that an empathetic way of communication generally does not lead to expectancy violations, regardless of the gender, since empathy does not offend that easily and hence has less potential to be perceived as violating, when compared to aggressive tonalities [e.g., (15, 23)].

Conversely, as second hypothesis, we expected an increase in young adults’ expectancy violations when they were exposed to an aggressive message by a female compared to an aggressive message by a male communicator. This, too, was rejected according to the present study. A possible explanation of this finding might be that young adults’ affective reactions due to aggressive communication styles may override effects of violations of expectancies regarding gender stereotypes, because the former is more intense. This finding might be also explained by the severity of the topic of COVID-19. Not only is COVID-19 a life threat for many individuals and has already caused millions of deaths, people additionally fear vaccination complications [e.g., (57)]. Thus, it seems that certain severe health threat topics have such heavy impacts that profane male–female heuristics move to the periphery of attention.

However, there was a main effect of an aggressive communication style on young adults’ expectancy violations unrelated to the gender of the communicator. Based on the evidence, it is apparent that aggressive communication styles of any communicator—female or male—might generally, lead to expectancy violations. This effect most likely occurs due to the fact that an aggressive communication style is unexpected and deemed as inappropriate especially coming from (health) experts [e.g., (37)]. Still, due to the severity of the COVID-19 pandemic, many experts seem to have become frustrated and turn to more urgent and aggressive ways of spreading health messages (14). According to our study, this cannot be advised, because, further, this study provided evidence that an aggressive communication style of health experts on social media was associated with decreased young adults’ perceived credibility of the communicators. This is in line with prior research [e.g., (17)] and could be dangerous for the whole society in times of the COVID-19 pandemic, because low credibility of the communicating health expert online could negatively affect young adults’ supportive attitudes toward COVID-19 vaccinations [e.g., (16, 24)].

With regard to hypothesis 4, there was no interaction effect of communication style and psychological distance. A possible interpretation of this finding could be that a global pandemic affects everyone worldwide and is not a specific phenomenon occurring in one spatial area only. In this case, an international health expert may be perceived as just as competent and trustworthy as a local one. Since COVID-19 is a matter of “life and death” and not of place of residence, local expertise and similarity may not be that important for young adults to accept recommendations of health experts on social media regarding COVID-19 [e.g., (21)].

For hypothesis 5, there was a significant effect showing association between higher levels of psychological distance and lower perceived credibility. This is in line with research by Citera et al. (58) who found that perceived levels of psychological distance negatively affected individuals’ perceptions of credibility. Credibility, however, is crucial in achieving persuasive communication goals [e.g., (16)]. This shows the importance of keeping young adults’ perceptions of psychological distance to the health expert online as low as possible for vaccination appeals on social media. Other cues on social media profiles that theoretically could heighten perceptions of psychological distance to health experts should be avoided to bypass potential negative effects backfiring on health experts’ credibility. Besides the origin of health experts, further work is required to test other social media cues that could theoretically increase perceived psychological distance.

Further, our data suggested that perceived credibility of the communicator is positively related to young adults’ supportive attitudes toward COVID-19 vaccination, supporting our sixth hypothesis. Previous literature investigating information processing has shown that non-experts tend to process information based on heuristics such as peripheral cues rather than on arguments [e.g., (34)]. Following these lines, young adults’—uncommon to be health experts—might tend to process information about the COVID-19 vaccination *via* the peripheral route using the perception of the credibility of the health expert as a heuristic [e.g., (34)]. Especially when it comes to online social media posts which are limited in their word count (i.e., Twitter), young adults might tend to rely on their impressions regarding the health experts’ credibility, since the short postings cannot possibly contain enough information for fact-based decisions.

To sum it up, the results of this study provided evidence that an aggressive communication style, in comparison to an empathetic communication style, violated the expectancies of how a medical professional should communicate. In turn, these violated expectancies were associated with lower perceived credibility of the communicator, which was further related to less supportive attitudes toward COVID-19 vaccination of the recipients of the message. In the context of COVID-19 vaccinations, the aggressive appeal coming from health professionals may be not expected by young adults. Additionally, some people may interpret vaccination appeals as an intrusion to their personal freedom in decision making that an aggressive tone leads to psychological reactance (59). When a person is told aggressively that they should get vaccinated against COVID-19, it may be perceived as inappropriate and limiting the own freedom to decide. Yet, aggressive communication addressing the necessity of a COVID-19 vaccination is currently increasing in the public scientific debate (14) with the goal to push supporting attitudes toward COVID-19 vaccinations, when in reality it may result in the opposite (12).

These findings once again highlighted the importance of source credibility in mediating health communication effects on attitudes toward vaccinations. Recent findings pointed into the same direction, showing that credibility by and large is crucial in achieving persuasive goals [e.g., (16)]. Especially within scientific debates, an aggressive communication tone is commonly perceived as less credible and could lead to decreased supportive attitudes toward COVID-19 vaccinations (24). Overall, this is in line with health communication research on scientific debates in general [e.g., (24)].

### 7.1. Limitations

A number of limitations of this study need to be considered. First, the majority of respondents in our sample indicated they were already vaccinated. The perceptions of the messages as well as the communicators might differ in a sample with only not-vaccinated participants. Second, the study was conducted in Australia. The findings are not generalizable to other countries as countries all over the world dealt slightly different with COVID-19 vaccinations. Australia, for instance, implemented a mandatory vaccination only for selected population groups [e.g., (60)]. The results of the study may not be applicable for countries with a mandatory vaccination for the whole population or for countries without an obligation to vaccinate against COVID-19. Third, while this study provided some valuable insights on self-reported attitudes, the actual behavior of the respondents can differ in reality. This especially is the case for sensitive topics such as health related topics dealing with support for vaccination, because they are associated with collective responsibility where social desirability could lead to biased responses from participants. Future studies investigating supportive attitudes toward COVID-19 vaccinations should additionally measure actual vaccination behavior of young adults. Moreover, due to our experimental method, we cannot draw any conclusions on possible long-term effects of the shown health messages on social media. Since, COVID-19 vaccinations are a topic that is strongly represented in media reporting, longitudinal research is needed to account for habituations effects of young adults with this kind of messages. Lastly, our research focused on the social media platform Twitter. Findings cannot be applied to any other social media platform due to the unique logic of Twitter (i.e., word limit). Besides text, future studies should include audio-visual elements on other social media platforms than Twitter.

## 8. Conclusion

To conclude, in debates about vaccination on social media, we often see and hear an aggressive tone, urging individuals to vaccinate. Our findings suggest that telling young adults to get vaccinated in an aggressive tone does not pay off at all. In contrast: communicating aggressively about COVID-19, independent of the health experts’ gender (female vs. male) and country of origin (local vs. international) was related to more violations of young adults’ expectancies compared to an empathetic communication style that urges COVID-19 vaccinations. Young adults’ expectancy violations were then associated with lower perceived credibility of the health expert and consequently, with lower support for COVID-19 vaccinations. In a nutshell, empathetic communication is clearly a better alternative when addressing young adults on social media.

## Data availability statement

The datasets presented in this study can be found in online repositories. The names of the repository/repositories and accession number(s) can be found at: https://osf.io/zv287/?view_only=69951be31a0d4d8e87ed245f7abefa5f.

## Ethics statement

The studies involving human participants were reviewed and approved by Institutional Review Board of the University of Vienna (approval ID: 20210714_055). Data collection was conducted by a professional polling company. The participants provided their written informed consent to participate in this study. For participants under the age of 18, the polling company was responsible for obtaining written informed consent to participate in this study from the participants’ legal guardian/next of kin.

## Author contributions

JK, SD, and ES contributed to the conception and design of the study. MS and JM organized the data collection. JM performed the statistical analysis. JK, SD, ES, MS, and AN wrote the sections of the manuscript. AN, JK, MS, and JM reviewed and revised the manuscript. All authors contributed to the article and approved the submitted version.

## Footnotes

^1^As an additional test, we performed bootstrapping with 95% bias-corrected confidence intervals in order to further bolster the credibility of our conclusions and confirm the robustness of our initial tests. Based on the results, our findings remain unchanged, with the exception of the association between aggressive communication style and psychological distance, which was found to be statistically non-significant based on bootstrapping. However, it is important to note that this non-significant association does not impact our overall conclusions. Moreover, we note that we did not perform additional *p* -value adjustments for the presented results due to the implementation of bootstrapping with 95% bias-corrected confidence intervals, which provides robust estimates and p-values. In this case, *p* -value adjustments could lead to overly conservative results, potentially increasing the risk of false negatives [e.g., (52)]. Furthermore, we would like to emphasize that we have made all study data publicly available (see Data Availability Statement), enabling researchers to independently replicate our study and verify the results, thereby ensuring the robustness and validity of our findings.
