# Supplementary material for: “I am Young, Why Should I Vaccinate?” How empathetic and aggressive communication on social media impact young adults’ attitudes toward COVID-19 vaccination
Source: Front Public Health. 2023 Oct 6;11:1190847. doi: 10.3389/fpubh.2023.1190847 (PMC10587396; doi:10.3389/fpubh.2023.1190847)
Supplement: Supplementary file 1 [file Data_Sheet_1.PDF]

Supplementary Material

"I am Young, Why Should I Vaccinate?" How Empathetic and Aggressive Communication on Social Media Impact Young Adults' Attitudes toward COVID-19 Vaccination

Jaroslava Kaňková*, Melanie Saumer, Ariadne Neureiter, Sofia Darovskikh, Elena Shargina, Jörg Matthes

*** Correspondence:**
Corresponding Author: jaroslava.kankova@univie.ac.at

1 Supplementary Data

Supplementary Data. Examples of stimuli used in this study. (1) Aggressive female international communicator; (2) Aggressive female international communicator; (3) Empathetic male local communicator; (4) Empathetic male local communicator

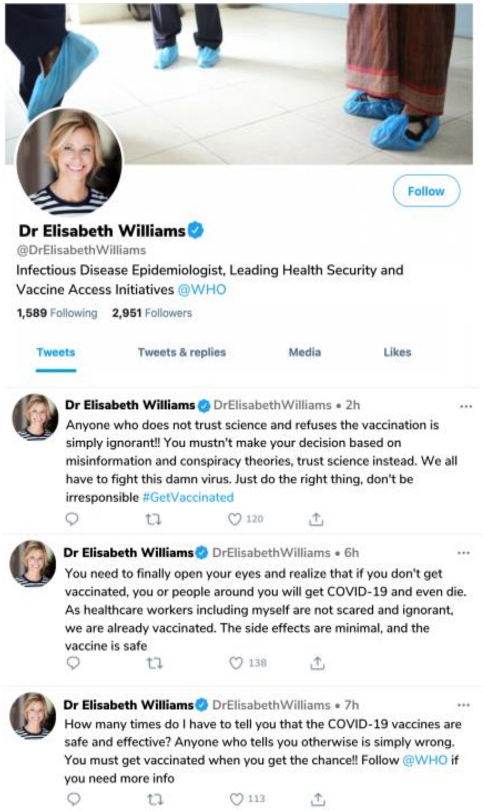
Follow
Dr Elisabeth Williams
@DrElisabethWilliams
Infectious Disease Epidemiologist, Leading Health Security and Vaccine Access Initiatives @WHO
1,589 Following 2,951 Followers
Tweets
Tweets & replies
Media
Likes
Dr Elisabeth Williams DrElisabethWilliams • 2h
Anyone who does not trust science and refuses the vaccination is simply ignorant!! You mustn't make your decision based on misinformation and conspiracy theories, trust science instead. We all have to fight this damn virus. Just do the right thing, don't be irresponsible #GetVaccinated
120
Dr Elisabeth Williams DrElisabethWilliams • 6h
You need to finally open your eyes and realize that if you don't get vaccinated, you or people around you will get COVID-19 and even die. As healthcare workers including myself are not scared and ignorant, we are already vaccinated. The side effects are minimal, and the vaccine is safe
138
Dr Elisabeth Williams DrElisabethWilliams • 7h
How many times do I have to tell you that the COVID-19 vaccines are safe and effective? Anyone who tells you otherwise is simply wrong. You must get vaccinated when you get the chance!! Follow @WHO if you need more info
113

(1)

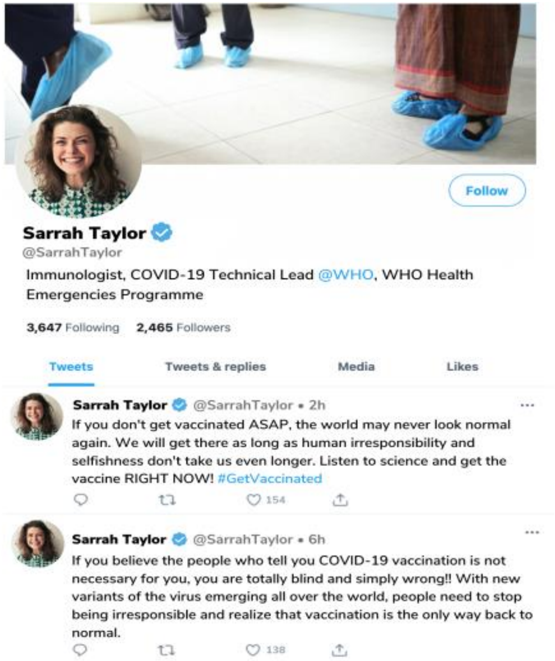
Follow
Sarrah Taylor
@SarrahTaylor
Immunologist, COVID-19 Technical Lead @WHO, WHO Health Emergencies Programme
3,647 Following 2,465 Followers
Tweets
Tweets & replies
Media
Likes
Sarrah Taylor @SarrahTaylor • 2h
If you don't get vaccinated ASAP, the world may never look normal again. We will get there as long as human irresponsibility and selfishness don't take us even longer. Listen to science and get the vaccine RIGHT NOW! #GetVaccinated
154
Sarrah Taylor @SarrahTaylor • 6h
If you believe the people who tell you COVID-19 vaccination is not necessary for you, you are totally blind and simply wrong!! With new variants of the virus emerging all over the world, people need to stop being irresponsible and realize that vaccination is the only way back to normal.
138

(2)

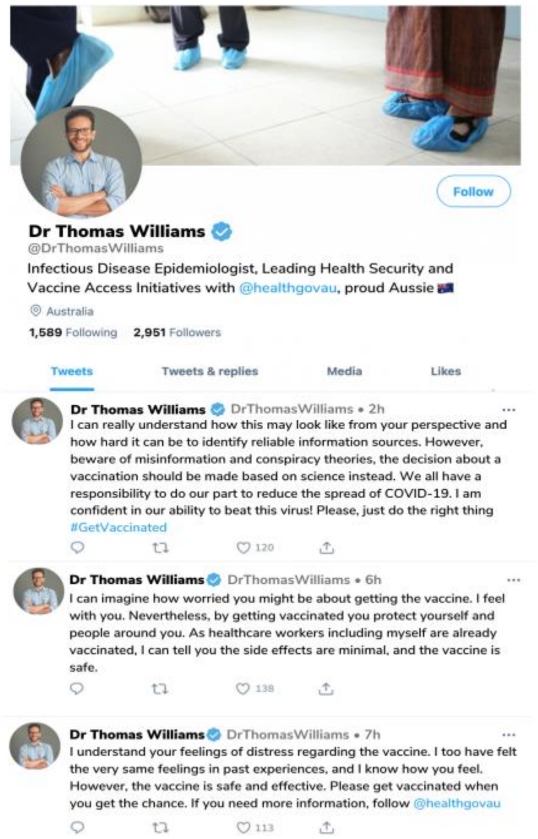
Follow
Dr Thomas Williams
@DrThomasWilliams
Infectious Disease Epidemiologist, Leading Health Security and Vaccine Access Initiatives with @healthgovau, proud Aussie
Australia
1,589 Following 2,951 Followers
Tweets
Tweets & replies
Media
Likes
Dr Thomas Williams DrThomasWilliams • 2h
I can really understand how this may look like from your perspective and how hard it can be to identify reliable information sources. However, beware of misinformation and conspiracy theories, the decision about a vaccination should be made based on science instead. We all have a responsibility to do our part to reduce the spread of COVID-19. I am confident in our ability to beat this virus! Please, just do the right thing #GetVaccinated
120
Dr Thomas Williams DrThomasWilliams • 6h
I can imagine how worried you might be about getting the vaccine. I feel with you. Nevertheless, by getting vaccinated you protect yourself and people around you. As healthcare workers including myself are already vaccinated, I can tell you the side effects are minimal, and the vaccine is safe.
138
Dr Thomas Williams DrThomasWilliams • 7h
I understand your feelings of distress regarding the vaccine. I too have felt the very same feelings in past experiences, and I know how you feel. However, the vaccine is safe and effective. Please get vaccinated when you get the chance. If you need more information, follow @healthgovau
113

(3)

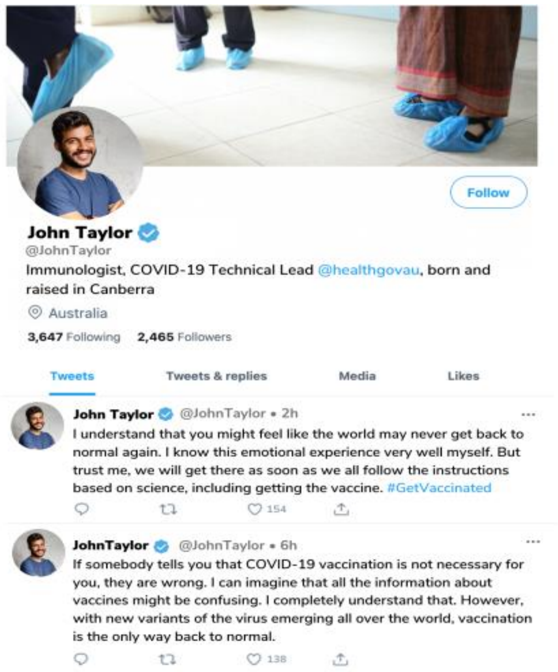
Follow
John Taylor
@JohnTaylor
Immunologist, COVID-19 Technical Lead @healthgovau, born and raised in Canberra
Australia
3,647 Following 2,465 Followers
Tweets
Tweets & replies
Media
Likes
John Taylor @JohnTaylor • 2h
I understand that you might feel like the world may never get back to normal again. I know this emotional experience very well myself. But trust me, we will get there as soon as we all follow the instructions based on science, including getting the vaccine. #GetVaccinated
154
JohnTaylor @JohnTaylor • 6h
If somebody tells you that COVID-19 vaccination is not necessary for you, they are wrong. I can imagine that all the information about vaccines might be confusing. I completely understand that. However, with new variants of the virus emerging all over the world, vaccination is the only way back to normal.
138

(4)

2 Supplementary Figures and Tables

2.1 Supplementary Figures

Supplementary Figure 1. Conceptual Framework

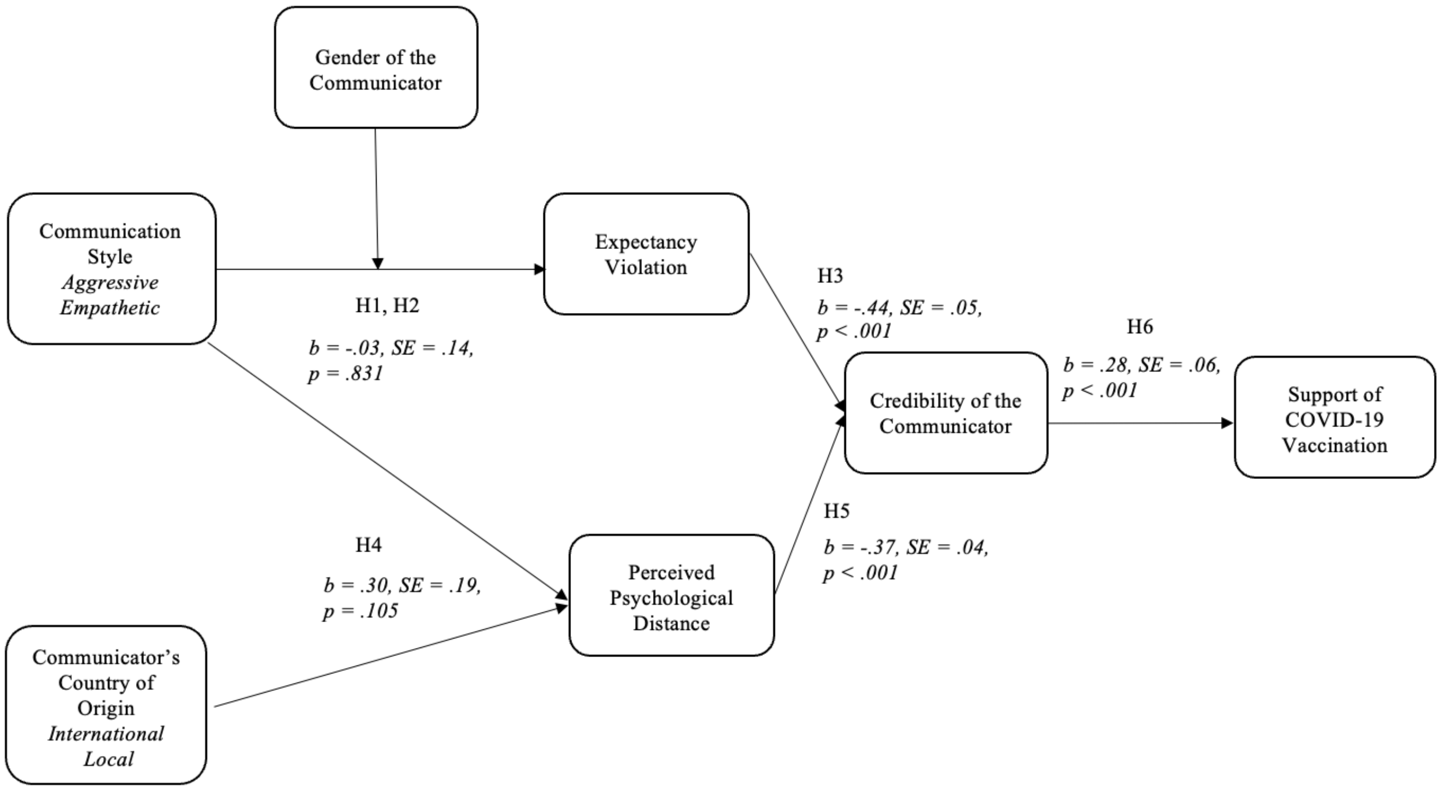

2.2 Supplementary Tables

Supplementary Table 1. Description of Measures

Variable	*Type of Scale*	*Items*	*Cronbach's α*	SE	95% CI	*M (SD)*
Expectancy Violation	Five-point Likert scale ('disagree strongly' to 'agree strongly')	"The communicator used an appropriate communication style for a medical expert" (*reversed coded*), "I don't think medical experts usually communicate this way", "The way the medical expert communicated did not surprise me" (*reversed coded*), "I expect medical experts to normally communicate this way" (*reversed coded*).	.71	.03	[0.65, 0.77]	2.83 (0.81)
Psychological Distance (all *reversed coded*)	Five-point Likert scale ('disagree strongly' to 'agree strongly')	"The communicators are similar to me", "The communicators think like I do", "The communicators and I have a lot in common", "The communicators probably behave like me".	.91	.01	[0.90, 0.93]	3.01 (1.02)

Perceived Source Credibility	Five-point semantic differential scale	‘Unfriendly – Friendly’, ‘Cold – Warm’, ‘Unsympathetic – Sympathetic’ (*warmth*); ‘Unintelligent – Intelligent’, ‘Incompetent – Competent’, ‘Unqualified – Qualified’ (*competence*); ‘Not Credible – Credible’, ‘Insincere – Sincere’, ‘Untrustworthy – Trustworthy’ (*trustworthiness*).	.94	.01	[0.93, 0.96]	3.56 (0.89)
Supportive Attitudes toward COVID-19 vaccination	Five-point Likert scale (‘disagree strongly’ to ‘agree strongly’)	“COVID-19 vaccines are a great scientific and medically safe invention that will allow us to overcome the pandemic”, “COVID-19 vaccines have some risks, but their overall cost benefit is positive”, “COVID-19 vaccines have many risks for different age groups, but they are necessary for escaping worse outcomes”.	.73	.03	[0.64, 0.79]	3.70 (0.88)

Political Orientation	Ten-point Likert scale (‘very left’ to ‘very right’)	“In politics people sometimes talk of “left” and “right”. Using this scale, where would you place yourself on this scale”.	5.67 (1.95)
Vaccination Status		“Have you been vaccinated against COVID-19 with at least one shot?”; 1. “Yes, I have received the first shot”; 2. “Yes, I am fully vaccinated”; 3. “No”	1.84 (0.70)
Prior COVID-19 Infection Experiences		“Have you been infected with COVID-19 in the past?”; 1. “Yes, and I have experienced some symptoms”; 2. “Yes, and I have not experienced any symptoms”; 3. “I think I was infected, but I did not get tested to confirm it”; 4. “No”.	3.63 (0.87)

Manipulation Check - Communicator's Gender		"The authors of the tweets were…"; 1. "Male"; 2. "Female"; 3. "It was not specified"; 4. "I don't know".	1.59 (0.60)
Manipulation Check – Communicator's Country of Origin		"Do you know which country were the communicators from?"; 1. "Australia"; 2. "UK"; 3. "USA"; 4. "Germany"; 5. "Italy"; 6. "China"; 7. "It was not specified"; 8. "I don't know".	5.04 (3.09)
Manipulation Check – Communication Style	Five-point Likert scale ('disagree strongly' to 'agree strongly')	"The communicators came across as aggressive"; "The communicators showed empathy"; "I felt like the communicator could relate to my feelings"; "I felt like the communicator understood how people feel towards the COVID-19 vaccination"; "I felt verbally attacked by the communicators"; "The communicators indicated that my opinions	3.10 0.58)

towards the vaccine can be ignorant and irresponsible".

Supplementary Table 2. Distribution of Participants in Experimental Conditions

Experimental Condition	*N*	*%*
Aggressive Male International	*47*	*11.5%*
Aggressive Male Local	51	12.4%
Aggressive Female International	60	14.6%
Aggressive Female Local	*50*	*12.2%*
Empathetic Male International	59	14.4%
Empathetic Male Local	45	11.0%
Empathetic Female International	51	12.4%
Empathetic Female Local	47	11.5%

Supplementary Table 3. Randomization Check

Variables	
	Experimental Conditions
Age	$F(7, 402) = 0.58$, $p = .771$
Gender	$\chi2(7) = 2.73$, $p = .130$
Education	$\chi2(7) = 26.53$, $p = .327$
Political orientation	$F(7, 402) = 0.56$, $p = .790$
Prior COVID-19 infections	$\chi2(7) = 9.45$, $p = .082$
Vaccination status	$\chi2(7) = 7.55$, $p = .031$

Note. No systematic differences for our control variables age, gender, education, political orientation and prior COVID-19 infections between our conditions, except for vaccination status

Supplementary Table 4. Manipulation Check

Condition			
Perception of Communication Style; M (SD)			
	Aggressive, Ignorant and Irresponsible	*Empathetic, Emotive, and Understanding*	*t*
Aggressive	**3.19 (0.91)**	3.05 (0.91)	$t(408) = 7.23, p < .001$[a]
Empathetic	2.49 (0.97)	**3.67 (0.81)**	$t(408) = -7.59, p < .001$[b]
Gender of the Health Expert; (Dummy Variable)			
	Perception of Female Health Expert		*χ2*
Female Health Expert	***n* = 197**		$\chi2(3) = 326.06, p < .001$
Male Health Expert	*n* = 12		
Origin of the Health Expert (Dummy Variable)			
	Perception of Internationality of Health Expert		*χ2*
International	***n* = 188**		$\chi2(1) = 54.88, p < .001$
Local	*n* = 29		

Note. [a]Compared to the empathetic condition (*n* = 202). [b]Compared to the aggressive condition (*n* = 208). Significantly highest means (*SD*) or frequencies are depicted in bold

Supplementary Table 5

Results of the Structural Equation Model

Predictors	Expectancy Violation		Psychological Distance		Credibility of the Communicator		Supportive Attitudes toward COVID-19 Vaccination	
	b	SE	b	SE	b	SE	b	SE
Age	-.01	.01	-.04	.02	-.00	.01	-.02	.01
aGender Participants (female)	.15	.07	.26**	.10	-.09	.07	.08	.09
aEducation (high)	-.02	.09	.01	.11	.05	.08	-.06	.10
Political Orientation (left-right)	-.05**	.02	-.05*	.02	-.06***	.02	.02	.02
aStatus of Vaccination	-.18	.09	-.52***	.12	-.21*	.09	.44***	.12
aPrior COVID-19 infections	-.06	.10	-.24	.13	-.14	.10	-.04	.12
aCommunicator Style (aggressive)	.56***	.13	.13**	.17	-.24	.12	.14	.16
aCommunicator Country (international)	-.05	.13	-.00	.16	-.11	.12	-.22	.15
aGender Communicator (male)	.16	.13	.08	.17	-.24	.12	.01	.15

Supplementary Table 5 (*Continued)*

Predictors	Expectancy Violation		Psychological Distance		Credibility of the Communicator		Supportive Attitudes toward COVID-19 vaccination	
	b	SE	b	SE	b	SE	b	SE
aCommunicator Style (aggressive) * aCommunicator gender (male)	-.03	.14	.14	.19	.08	.14	.07	.17
aCommunicator Style (aggressive) * aCommunicator Country (international)	.13	.14	.30	.19	-.11	.14	.08	.17
aCommunicator Country (international) * aCommunicator gender (male)	-.08	.14	-.26	.19	.33*	.14	.27	.17
Expectancy Violation					-.44***	.05	.05	.07
Psychological Distance					-.37***	.04	-.20***	.05
Credibility of the Communicator							.28***	.06
R2	.19		.14		.44		.22	

Note. *N* = 410; **p* < .05, ***p* < .01, ****p* < .001; $_a$dummy coded variables.

Supplementary Table 6. Overview of the Hypotheses

No.	Hypotheses	Status
H1	An aggressive message by a female will lead to higher expectancy violation compared to the same message by a male.	Rejected
H2	An empathetic message by a male will lead to higher expectancy violation compared to the same message by a female.	Rejected
H3	Expectancy violation is negatively related to perceived credibility of the communicator.	Accepted
H4	An (a) aggressive and (b) empathetic message by an international communicator will lead to higher psychological distance compared to the same message by a local one.	Rejected
H5	Psychological distance is negatively related to perceived credibility of the communicator.	Accepted
H6	Perceived credibility of the communicator is positively related to supportive attitudes toward COVID-19 vaccination.	Accepted
